# Very little influenza in the WHO European Region during the 2020/21 season, weeks 40 2020 to 8 2021

**DOI:** 10.2807/1560-7917.ES.2021.26.11.2100221

**Published:** 2021-03-18

**Authors:** Cornelia Adlhoch, Piers Mook, Favelle Lamb, Lisa Ferland, Angeliki Melidou, Andrew J Amato-Gauci, Richard Pebody, the European Influenza Surveillance Network, European Influenza Surveillance Network, European Influenza Surveillance Network, Romella Abovyan, Shushan Sargsyan, Monika Redlberger-Fritz, Inna Karaban, Natallia Shmialiova, Nathalie Bossuyt, Isabelle Thomas, Nina Rodić-Vukmir, Amela Dedeić-Ljubović, Goranka Petrović, Irena Tabain, Despo Pieridou, Christos Karagiannis, Helena Jirincova, Jan Kyncl, Lasse Skafte Vestergaard, Ramona Trebbien, Olga Sadikova, Liidia Dotsenko, Niina Ikonen, Outi Lyytikäinen, Vincent Enouf, Yu Jin Jung, Silke Buda, Ralf Dürrwald, Georgia Gioula, Thanos Kossyvakis, Zsuzsanna Molnár, Mónika Rózsa, Gudrun Aspelund, Arthur Löve, Lisa Domegan, Linda Dunford, Zalman Kaufman, Michal Mandelboim, Antonino Bella, Simona Puzelli, Tleumbetova Nazym, Usserbayev Aidar, Raina Nikiforova, Gatis Pakarna, Greta Gargasiene, Svajūnė Muralytė, Joël Mossong, Tamir Abdelrahman, Jackie Maistre Melillo, Tanya Melillo, Alina Druc, Mariana Apostol, Adam Meijer, Ron A.M. Fouchier, Bosevska Golubinka, Dragan Kochinski, Trine Hessevik Paulsen, Olav Hungnes, Raquel Guiomar, Ana Paula Rodrigues, Rodica Popescu, Odette Popovici, Anna Sominina, Daria Danilenko, Dragana Dimitrijevic, Maja Sočan, Katarina Prosenc, Francisco Pozo Sanchez, Concepción Delgado-Sanz, Emma Byström, AnnaSara Carnahan, Ana Rita Gonçalves, Rita Born, Emine Avci, Ayse Basak Altas, Iryna Demchyshyna, Tetiana Dykhanovska, Mary Sinnathamby, Jamie Lopez Bernal

**Affiliations:** 1 European Centre for Disease Prevention and Control (ECDC), Stockholm, Sweden; 2 World Health Organization (WHO) Regional Office for Europe, Copenhagen, Denmark; 3 The members of the European Influenza Surveillance Network are listed below

**Keywords:** Europe, influenza, surveillance, epidemiology, 2020/21 season

## Abstract

Between weeks 40 2020 and 8 2021, the World Health Organization European Region experienced a 99.8% reduction in sentinel influenza virus positive detections (33/25,606 tested; 0.1%) relative to an average of 14,966/39,407 (38.0%; p < 0.001) over the same time in the previous six seasons. COVID-19 pandemic public health and physical distancing measures may have extinguished the 2020/21 European seasonal influenza epidemic with just a few sporadic detections of all viral subtypes. This might possibly continue during the remainder of the influenza season.

We study features of influenza epidemiology in the World Health Organization (WHO) European Region from week 40 2020 to week 8 2021, a period when in usual seasons the highest influenza activity (peak of seasonal epidemic) would be expected. Results are compared to those of the previous six seasons (2014/15–2019/20).

## Influenza surveillance in the WHO European Region

Influenza surveillance in the WHO European Region is jointly coordinated by the European Centre for Disease Prevention and Control (ECDC) and the WHO Regional Office for Europe [1,2]. Countries and territories report weekly to The European Surveillance System (TESSy) syndromic and/or virological data. The data originate from sentinel primary care sites (using case definitions either for influenza-like illness (ILI) or acute respiratory infection (ARI) [1]), designated hospital sites (for laboratory-confirmed influenza hospitalisation or cases of severe ARI (SARI)), as well as non-sentinel settings, such as those in the context of outbreaks, primary care diagnostic laboratories, hospitals and other healthcare facilities. Reported virological data include information on circulating virus types, subtypes and lineages. In sentinel surveillance, the number of tested specimens is used as denominator to calculate influenza virus positivity, which is one indicator for influenza activity and intensity. Data are published weekly on the FluNewsEurope webpage [1].

## The COVID-19 pandemic and influenza in 2020

In late 2019, severe acute respiratory syndrome coronavirus 2 (SARS-CoV-2), which is responsible for coronavirus disease (COVID-19), emerged in China [3], from where it further spread. This subsequently led to a pandemic declared on 11 March 2020 [4], which is still ongoing. The implementation during the pandemic of strict public health measures (e.g. working from home, school closures, limiting social gatherings, increased hygiene measures, wearing masks etc.) to decrease SARS-CoV-2 transmission also reduced the circulation of other respiratory viruses [5,6]. This was reflected by an all-time low level of influenza activity in the southern hemisphere in 2020 and elsewhere [7-10].

**Figure 1:**
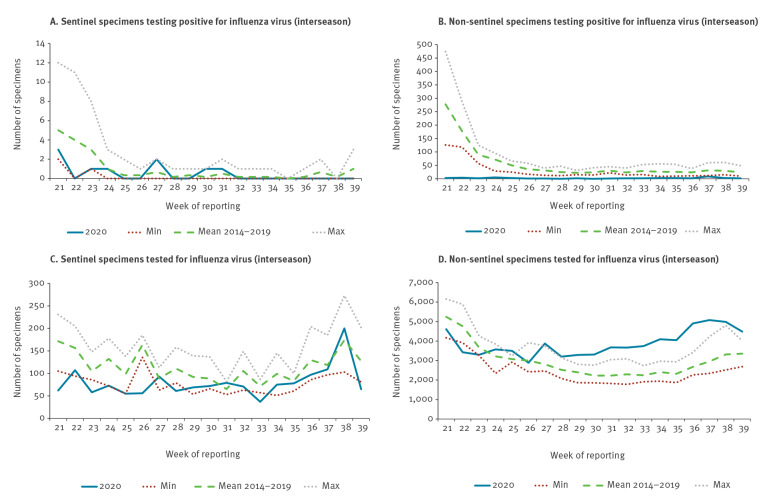
Numbers of specimens in the 2020 interseason (weeks 21–39) (A) testing positive for influenza virus in sentinel surveillance and (B) in non-sentinel surveillance, as well as (C–D) numbers of specimens tested for influenza virus by these respective surveillance systems, compared with minimum, mean and maximum of 2014–2019 interseasons, WHO European Region

For the WHO European Region, in autumn 2020, we predicted unusually late and low level influenza activity for the 2020/21 winter, based on the low numbers of specimens testing positive for influenza virus, detected in the summer months by sentinel and non-sentinel surveillance (Figure 1A,B), despite substantial testing for influenza virus during the COVID-19 pandemic (Figure 1C,D) [1,11].

Max: maximum; min: minimum; WHO: World Health Organization.

Please note the different y-axis scales.

## The 2020/21 season in the WHO European Region

We further describe the total numbers of influenza detections and numbers of specimens tested for influenza virus from sentinel, non-sentinel and hospital surveillance systems in the Region from week 40 2020 to week 8 2021. Results are compared to the averages, minimums and maximums of the previous six seasons (2014/15–2019/20). We used a paired t-test with a significance level p = 0.05.

### Sentinel surveillance results

**Figure 2:**
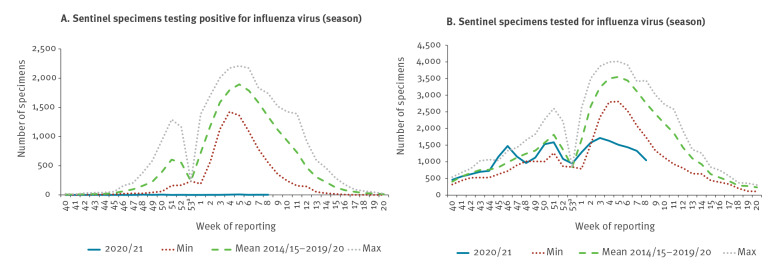
Sentinel-surveillance-obtained numbers of (A) specimens testing positive for influenza virus and (B) specimens tested in the 2020/21 season up to week 8, compared with minimum, mean and maximum of previous seasons (weeks 40–20) in 2014/15–2019/20, WHO European Region

Between weeks 40 2020 and 8 2021, 37 countries and territories in the WHO European Region tested 25,606 sentinel specimens, of which 33 tested positive for influenza virus, with both virus types A and B detected in 11 countries (Figure 2A). Among type A viruses, 13 influenza A(H1)pdm09 viruses were detected compared with six A(H3) and one type A unsubtyped; two of 13 type B viruses detected were ascribed to a lineage, B/Victoria. This number of specimens testing positive is 99.8% lower than in any previous comparable period (first 22 weeks of the 2014/15–2019/20 season) when an average of 14,966 specimens tested positive (range: 12,176–18,748).

Max: maximum; min: minimum; WHO: World Health Organization.

^a^ Only seasons 2015/16 and 2020/21 had week 53.

Please note the different y-axis scales.

The level of tested sentinel specimens in 2020/21 was similar to prior seasons between weeks 40 2020 and 2 2021, but lower from week 3 2021 (Figure 2B). Compared with previous seasons and respective time periods, the overall number of tested sentinel specimens were lower than the average of 39,407 specimens from 2014/15 to 2019/20 (range: 32,746–45,124; p < 0.01).

**Figure 3:**
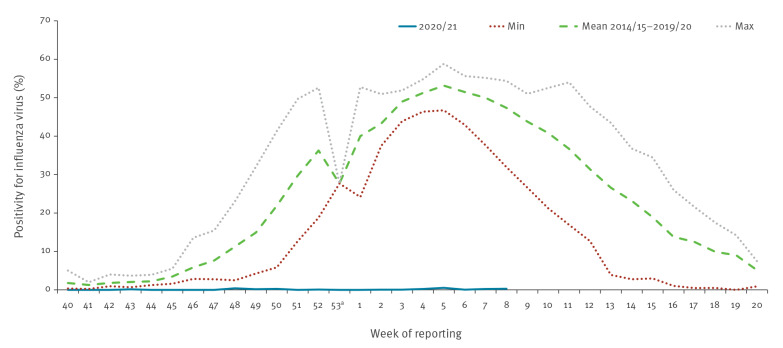
Proportion of specimens testing positive (positivity) for influenza virus in sentinel surveillance in weeks 40 2020–8 2021 compared with minimum, mean and maximum of previous seasons in 2014/15–2019/20, WHO European Region

A significantly lower positivity of 0.1% (33/25,606) compared with an average positivity of 38% (14,966/39,407) between week 40 and week 8 (p < 0.001) was estimated. The observed 0.3% (3/1,045) positivity in week 8 2021 indicated an exceptional reduction in activity compared with an expected positivity of 48.6% (1,340/2,755) for week 8, based on the average in previous seasons (Figure 3).

Max: maximum; min: minimum; WHO: World Health Organization.

^a^ Only seasons 2015/16 and 2020/21 had week 53.

### Non-sentinel surveillance results

**Figure 4:**
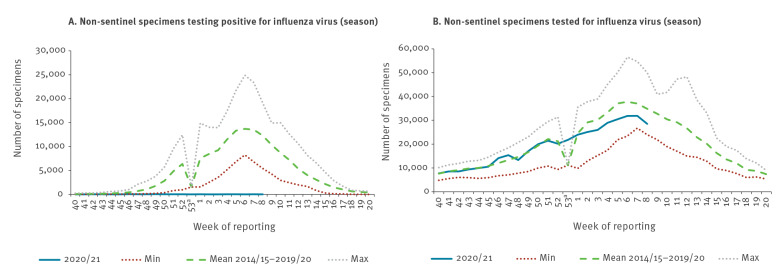
Non-sentinel-surveillance-obtained numbers of (A) specimens testing positive for influenza virus and (B) specimens tested in the 2020/21 season up to week 8, compared with minimum, mean and maximum of previous seasons (weeks 40–20) in 2014/15–2019/20, WHO European Region

By week 8 2021, 35 countries and territories tested 424,541 specimens from non-sentinel sources of which 679 specimens tested positive for influenza virus (Figure 4A). The positive specimens were reported from 19 countries and territories across the WHO European Region; the United Kingdom (UK) reported the majority of cases (488; 72%). Similar numbers of type A and B viruses were reported (343 vs 336). Among influenza A viruses, 278 were unsubtyped, 28 were A(H1)pdm09 and 37 A(H3). Among B viruses, 328 had no lineage determined, six were B/Victoria, and two B/Yamagata. Thus, the majority (89%; 606/679) of viruses was reported without subtype or lineage. The number of non-sentinel influenza virus detections, similar to sentinel detections, is 99.4% lower than the average of 117,777 specimens testing positive observed during comparable time periods in previous seasons (range: 63,409–165,375; p < 0.001; Figure 4A). The number of tested specimens from non-sentinel sources falls in the expected range when compared with similar time periods over the last six seasons (average: 469,126; range: 275,295–604,420; p = 0.15) (Figure 4B).

Max: maximum; min: minimum; WHO: World Health Organization.

^a^ Only seasons 2015/16 and 2020/21 had week 53.

Please note the different y-axis scales.

### Severity/hospitalisation

The substantially lower than expected circulation of influenza viruses this season resulted in a small number of reported laboratory-confirmed influenza hospitalisations. Until week 8 2021, only Ukraine reported nine hospitalised influenza cases in non-intensive care units (non-ICU) and two countries (Ukraine and England) reported two and nine hospitalised influenza cases in ICU, respectively.

**Figure 5:**
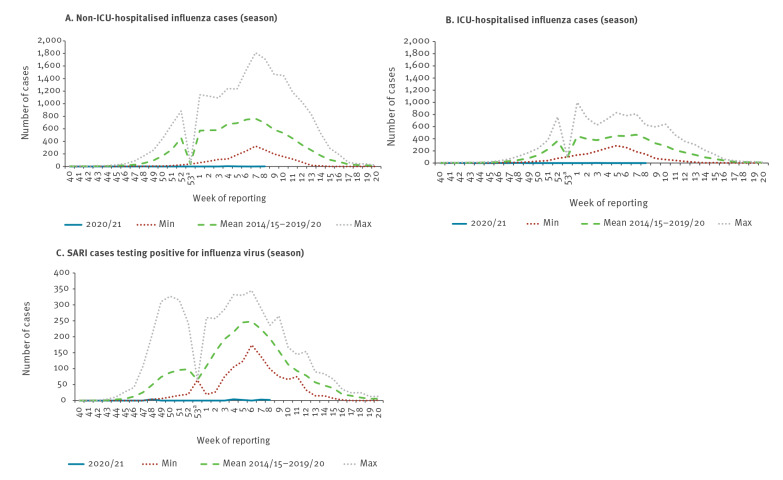
Number of hospitalised influenza cases in (A) non-ICU units, (B) ICU wards and (C) number of influenza positive cases from SARI surveillance, weeks 40 2020–8 2021 compared with minimum, mean and maximum of seasons 2014/15–2019/20, WHO European Region

ICU: intensive care unit; max: maximum; min: minimum; SARI: severe acute respiratory infection; WHO: World Health Organization.

^a^ Only seasons 2015/16 and 2020/21 had week 53.

In contrast to these nine non-ICU and 11 ICU cases, in previous seasons until the same time point, between 1,991 and 13,849 non-ICU and between 2,844 and 7,497 ICU cases with laboratory-confirmed influenza were reported by five to 11 countries and nine to 16 countries, respectively. The numbers obtained so far in the 2020/21 season represent 99.9% (non-ICU) and 99.8% (ICU) reductions in case numbers compared to the respective averages of 6,904 (non-ICU) and 4,602 (ICU) cases for the same period in prior seasons (p < 0.001; Figure 5A and B).

Please note the different y-axis scales.

From SARI-based surveillance, 13 non-EU/EEA countries and territories tested 8,446 SARI cases for influenza and only 15 cases (0.2%) were reported positive, 11 from Ukraine (eight A(H1)pdm09 and three type A unsubtyped), three from Armenia (all A(H3N2)) and one from Azerbaijan (type B virus). Significantly more SARI cases were tested for influenza this season compared with the average of 7,213 (range: 4,358–8,733; p = 0.02) during the same time period over the last six seasons, when 2,175 (30.2%; range: 1,098–3,212) tested positive. Despite increased testing, a 99.3% reduction in influenza cases was observed during the current season.

Whereas in primary care sentinel, non-sentinel and SARI-derived specimens, a mixture of all subtypes and lineages (albeit with limited B/lineage testing) was detected, in laboratory-based hospital surveillance, more influenza A, particularly A(H1)pdm09, was observed: in non-ICU, seven of the nine type A viruses were subtyped as A(H1)pdm09, while in ICU all 11 specimens were type A viruses, one subtyped as A(H1)pdm09 [1]. The low number of detections overall limits the interpretation of these findings.

## Discussion

During the annual seasonal epidemics in non-pandemic years, influenza causes high burden on the population and healthcare systems of the WHO European Region [12]. The COVID-19 pandemic through 2020 and 2021 had a large impact on influenza circulation in the WHO European Region as well as globally [7-10]. This 2020/21 influenza season is exceptional since the creation of the Global Influenza Surveillance and Response System (GISRS) network in 1952 [13,14]. Despite countries testing a high number of specimens for influenza, most likely in parallel to SARS-CoV-2, e.g. using multiplex assays, only sporadic influenza virus detections of all types, subtypes, and lineages have been reported in the WHO European Region. The epidemic threshold of 10% positivity has not been reached in the Region, and seasonal influenza as well as ILI and ARI (data not shown) detections were lower than those observed even during regular summer months. Countries have made large efforts to minimise the impact of the ongoing COVID-19 pandemic on influenza surveillance. In the past, vaccination efforts and communication campaigns on individual behaviour to minimise influenza have been conducted with limited success in reducing virus circulation, however, no strict measures like school closures, stay home orders, using masks, and general reduction of population movement globally, regionally and locally have been in place during any influenza season since 1918 [15]. Because of the lower reproduction number (R0) of influenza virus compared with SARS-CoV-2, such measures are likely to explain the much stronger impact on influenza circulation [16].

Sentinel surveillance is considered the gold standard to monitor influenza activity and to provide the highest data quality from a defined outpatient population (ILI or ARI) tested in National Influenza Centres (NICs) that also perform detailed virus characterisation analyses. In the current 2020/21 season, most of the samples testing positive for influenza are derived from non-sentinel specimens (collected from different sources without being based on a specific clinical case definition) likely being sampled for parallel SARS-CoV-2 testing. While data from non-sentinel surveillance is usually of a lower quality, e.g. most viruses are not subtyped/lineage determined, the broader specimen sample from non-sentinel surveillance has provided useful data in the situation of a pandemic that is not caused by influenza, where sentinel surveillance alone is not able to identify as many influenza cases because of the likely changes in healthcare seeking and referral behaviour as well as the overall lower numbers of tests performed.

Only four influenza viruses have been genetically characterised this season, and no viruses have been antigenically characterised, providing limited information for vaccine composition decisions [1]. Because of the limited circulation of influenza viruses, NICs have been challenged to collect representative specimens for influenza virus detection and subsequent virus characterisations [17]. Countries have been encouraged to better utilise non-sentinel specimens for subtype/lineage determination and virus characterisation.

## Conclusion

The coincidental reduction of influenza transmission due to COVID-19 measures within the WHO European Region will possibly continue, with very low-level circulation of influenza viruses for the rest of the 2020/21 season, as long as non-pharmaceutical public health and physical distancing measures remain in place.

## Members of the European Influenza Surveillance Network

**Armenia:** Romella Abovyan - Head of the Department of Epidemiology of Infectious and Noncommunicable Diseases, National Center for Disease Control and Prevention, Ministry of Health, Republic of Armenia. rabovyan75@gmail.com; Shushan Sargsyan - Head of Virology Laboratory Reference Laboratory Center of the National Center for Disease Control and Prevention, Ministry of Health, Republic of Armenia. premier_h@yahoo.com; **Austria:** Monika Redlberger-Fritz, Center for Virology, Medical University Vienna, Vienna, Austria, monika.redlberger@meduniwien.ac.at; **Belarus:** Inna Karaban, Department of Hygiene, Epidemiology and Prevention, Ministry of Health, Minsk, Belarus - National focal point for influenza - inna_kia@list.ru; Natallia Shmialiova, Head of Influenza laboratory and ILI, Republican Research and Practical Center for Epidemiology and Microbiology Minsk, Belarus - National focal point for influenza Virology shmelevanataliya@mail.ru; **Belgium:** Nathalie Bossuyt, Sciensano, Brussels, Belgium; Nathalie.Bossuyt@sciensano.be; Isabelle Thomas Sciensano, Brussels, Belgium; Isabelle.Thomas@sciensano.be; **Bosnia and Herzegovina:** Nina Rodić - Vukmir, Public Health Institute of Republic of Srpska. nina.rodic@gmail.com; Amela Dedeić-Ljubović, Clinical microbiology, Clinical Center University of Sarajevo, Sarajevo, Bosnia and Herzegovina; amela.ljubovic@hotmail.com; **Croatia:** Goranka Petrović, Infectious Diseases Epidemiology Service, Croatian Institute of Public Health, Zagreb, Croatia, goranka.petrovic@hzjz.hr; Irena Tabain, National Influenza Centar, Croatian Institute of Public Health, Zagreb,Croatia, irena.tabain@hzjz.hr; **Cyprus:** Despo Pieridou, National Influenza Centre, Microbiology Department, Nicosia General Hospital, Nicosia, Cyprus; dpieridou@mphs.moh.gov.cy; Christos Karagiannis, National Influenza Centre, Microbiology Department, Nicosia General Hospital, Nicosia, Cyprus; ckaragiannis@mphs.moh.gov.cy; **Czechia:** Helena Jirincova, National Institute of Public Health, Prague, Czech Republic; helena.jirincova@szu.cz; Jan Kyncl, National Institute of Public Health, Prague, Czech Republic; jan.kyncl@szu.cz; **Denmark:** Lasse Skafte Vestergaard, Infectious Disease Epidemiology and Prevention, Statens Serum Institut, Copenhagen, Denmark; LAV@ssi.dk; Ramona Trebbien, National Influenza Center, Statens Serum Institut, Copenhagen, Denmark; RATR@ssi.dk; **Estonia:** Olga Sadikova, Department of Infectious Diseases, Health Board, Influenza Centre Tallinn Estonia; olga.sadikova@terviseamet.ee; Liidia Dotsenko, Labour of Infectious Diseases, Health Board, Tallinn, Estonia; liidia.dotsenko@terviseamet.ee; **Finland:** Niina Ikonen, Expert Microbiology Unit, Department of Health Security, Finnish Institute for Health and Welfare (THL), Helsinki, Finland, niina.ikonen@thl.fi; Outi Lyytikäinen, Infectious Disease Control and Vaccinations Unit, Department of Health Security, Finnish Institute for Health and Welfare (THL), Helsinki, Finland, outi.lyytikainen@thl.fi; **France:** Vincent Enouf, Centre national de référence Virus des infections respiratoires, dont la grippe, CNRS-UMR 3569, Institut Pasteur, Paris, France; vincent.enouf@pasteur.fr; Yu Jin Jung, Santé Publique France (SpFrance), the French National Public Health Agency, St Maurice, France; YuJin.JUNG@santepubliquefrance.fr; **Germany:** Silke Buda, Robert Koch Institute, Berlin, Germany; budas@rki.de; Ralf Dürrwald, Robert Koch Institute, Berlin, Germany, DuerrwaldR@rki.de; **Greece:** Georgia Gioula, Aristotle University of Thessaloniki; National Influenza Reference Laboratory for N. Greece, Thessaloniki, Greece; ggioula@auth.gr; Thanos Kossyvakis, NIC ATHENS, Hellenic Pasteur Institute, Athens, Greece; akossivakis@pasteur.gr; **Hungary:** Zsuzsanna Molnár, National Public Health Center, Hungary; Department of Communicable Disease Epidemiology and Infection Control; molnar.zsuzsanna@nnk.gov.hu; Mónika Rózsa, National Public Health Center, Hungary; National Influenza Reference Laboratory; rozsa.monika@nnk.gov.hu; **Iceland:** Gudrun Aspelund, The Directorate of Health, Reykjavik, Iceland; gudruna@landlaeknir.is; Arthur Löve, Landspitali, University of Iceland, Reykjavik, Iceland; arthur@landspitali.is; **Ireland:** Lisa Domegan, Health Service Executive, Health Protection Surveillance Centre, Dublin, Ireland. lisa.domegan@hse.ie; Linda Dunford, National Virus Reference Laboratory, University College Dublin, Dublin, Ireland. linda.dunford@ucd.ie; **Israel:** Zalman Kaufman, Israel Center for Disease Control, Ministry of Health, Israel. Email: zalman.kaufman@moh.gov.il; Michal Mandelboim, Central Virology Laboratory Ministry of Health, Israel. Email: michalman@sheba.gov.il; **Italy:** Antonino Bella, Department of Infectious Diseases, Istituto Superiore di Sanità (National Institute of Health), Rome, Italy; antonino.bella@iss.it; Simona Puzelli, Department of Infectious Diseases, Istituto Superiore di Sanità (National Institute of Health), Rome, Italy; simona.puzelli@iss.it; **Kazakhstan:** Tleumbetova Nazym, National Reference Laboratory, Almaty, Kazakhstan; nn_nazym@mail.ru; Usserbayev Aidar, National Reference Laboratory, Almaty, Kazakhstan; dest_0294@mail.ru; **Latvia:** Raina Nikiforova, Centre for Disease Prevention and Control, Riga, Latvia; raina.nikiforova@spkc.gov.lv; Gatis Pakarna, Riga East University Hospital, Latvia, gatis.pakarna@aslimnica.lv; **Lithuania:** Greta Gargasiene, Chief Specialist of the Communicable Disease Management Division, National Public Health Center under the Ministry of Health, Lithuania. Email: greta.gargasiene@nvsc.lt; Svajūnė Muralytė, Head of Virological testing subdepartment of Clinical Testing Department, National public health surveillance laboratory, Lithuania. Email: svajune.muralyte@nvspl.lt; **Luxembourg:** Joël Mossong, Health Directorate, Luxembourg; joel.mossong@ms.etat.lu; Tamir Abdelrahman, National Health Laboratory, Luxembourg; tamir.abdelrahman@lns.etat.lu; **Malta:** Jackie Maistre Melillo, Infectious Disease prevention and Control unit, Malta; jackie.m.melillo@gov.mt; Tanya Melillo; Infectious Disease prevention and Control unit, Malta Tanya.melillo@gov.mt; **Moldova:** Alina Druc, Epidemiological surveillance of influenza and viral respiratory infections, National Agency for Public Health, alina.druc@ansp.gov.md; Mariana Apostol, Virological laboratory, National Agency for Public Health, mariana.apostol@ansp.gov.md; **The Netherlands:** Adam Meijer, National Institute for Public Health and the Environment, Bilthoven, the Netherlands, adam.meijer@rivm.nl; Ron A.M. Fouchier, Department of Viroscience, Erasmus MC, Rotterdam, the Netherlands, r.fouchier@erasmusmc.nl; **North Macedonia:** Bosevska Golubinka, Institute of Public Health, Skopje, golubinka@yahoo.com; Dragan Kochinski, Institute of Public Health of RN Macedonia, Skopje, North Macedonia; dragan.kocinski@gmail.com; **Norway:** Trine Hessevik Paulsen, Section for Respiratory, Blood-borne and Sexually transmitted infections, Department of Infection Control and Vaccines, Norwegian Institute of Public Health, Oslo, Norway; TrineHessevik.Paulsen@fhi.no; Olav Hungnes, Department of Virology, Section for Influenza and other respiratory viruses, Norwegian Institute of Public Health, Oslo, Norway; Olav.Hungnes@fhi.no; **Portugal:** Raquel Guiomar, National Reference Laboratory for Influenza and Other Respiratory Viruses, National Institute of Health Doutor Ricardo Jorge, Lisbon, Portugal; raquel.guiomar@insa.min-saude.pt; Ana Paula Rodrigues, Epidemiology, National Institute of Health Doutor Ricardo Jorge, Lisbon, Portugal; ana.rodrigues@insa.min-saude.pt; **Romania:** Rodica Popescu, National Institute of Public Health Romania - National Centre for Communicable Diseases Surveillance and Control, Bucharest, Romania; rodica.popescu@insp.gov.ro; Odette Popovici, National Institute of Public Health Romania - National Centre for Communicable Diseases Surveillance and Control, Bucharest, Romania; odette.popovici@insp.gov.ro; **Russian Federation:** Anna Sominina, Smorodintsev Research Institute of Influenza, National Influenza Centre Saint Petersburg; anna.sominina@influenza.spb.ru; Daria Danilenko, Smorodintsev Research Institute of Influenza, National Influenza Centre Saint Petersburg - daria.baibus@gmail.com; **Serbia:** Dragana Dimitrijevic, National coordinator for epidemiological influenza surveillance, Institute of Public Health of Serbia, Belgrade, Serbia; dragana.dimitrijevic@yahoo.com; Dragana Dimitrijevic dragana_dimitrijevic@batut.org.rs; **Slovenia:** Maja Sočan, National Institute of Public Health, Slovenia; Maja.Socan@nijz.si; Katarina Prosenc, National Laboratory for Health, Environment and Food, Slovenia; katarina.prosenc.trilar@nlzoh.si; **Spain:** Francisco Pozo Sanchez; Institute of Health Carlos III, Madrid, Spain. pacopozo@isciii.es; Concepción Delgado-Sanz. National Epidemiology Centre, CIBER de Epidemiología y Salud Pública (CIBERESP), Institute of Health Carlos III, Madrid, Spain. cdelgados@isciii.es; **Sweden:** Emma Byström, The Public Health Agency of Sweden, Solna, Sweden; emma.bystrom@folkhalsomyndigheten.se; AnnaSara Carnahan, Unit for Vaccination Programmes, Department of Communicable Disease Control and Health Protection, Solna, Sweden; Annasara.Carnahan@folkhalsomyndigheten.se; **Switzerland:** Ana Rita Gonçalves, National Reference Centre of Influenza, Laboratory of Virology, Geneva University Hospitals, Geneva, Switzerland; agnv@hcuge.ch; Rita Born, Federal Office of Public Health, Berne, Switzerland Rita.Born@bag.admin.ch; **Turkey:** Emine AVCI, Public Health General Directorate Department of Communicable Diseases Influenza Surveillance Section, Ankara, Turkey; emineicva@hotmail.com, emine.avci10@saglik.gov.tr; Ayse Basak ALTAS, Public Health General Directorate Department of National Microbiology and Biological Products Reference Laboratory National İnfluenza Center, Ankara, Turkey; aysebasakdemir@gmail.com; **Ukraine:** Iryna Demchyshyna, National Coordinator for Influenza for virological issues, State Institution “Public health Center of the Ministry of health of Ukraine”, i.demchyshyna@phc.org.ua; irad@i.ua; Tetiana Dykhanovska, National Coordinator for Influenza for epidemiological issues, State Institution “Public health Center of the Ministry of health of Ukraine”, t.dykhanovska@phc.org.ua; **UK:** Mary Sinnathamby, Public Health England - mary.sinnathamby@phe.gov.uk; Jamie Lopez Bernal, Public Health England - jamie.lopezbernal2@phe.gov.uk.

## References

[1] European Centre for Disease Prevention and Control (ECDC) and World Health Organization (WHO) Regional Office for Europe. Flu News Europe - Joint ECDC-WHO/Europe weekly influenza update. Stockholm and Copenhagen: ECDC and WHO/Europe; 2021. [Accessed: 10 Feb 2021]. Available from: https://flunewseurope.org/

[2] Snacken R Brown C. New developments of influenza surveillance in Europe. Euro Surveill. 2015;20(4):21020. 10.2807/ese.20.04.21020-en 25655056

[3] Tan W Zhao X Ma X Wang W Niu P Xu W A novel coronavirus genome identified in a cluster of pneumonia cases—Wuhan, China 2019− 2020. China CDC Weekly. 2020;2(4):61-2. 10.46234/ccdcw2020.017 PMC8393069 34594763

[4] World Health Organization (WHO). WHO Director-General's opening remarks at the media briefing on COVID-19 - 11 March 2020. Geneva: WHO; 2020. [Accessed 9 May 2020]. Available from: https://www.who.int/dg/speeches/detail/who-director-general-s-opening-remarks-at-the-media-briefing-on-covid-19---11-march-2020

[5] Spiteri G Fielding J Diercke M Campese C Enouf V Gaymard A First cases of coronavirus disease 2019 (COVID-19) in the WHO European Region, 24 January to 21 February 2020. Euro Surveill. 2020;25(9). 10.2807/1560-7917.ES.2020.25.9.2000178 32156327 PMC7068164

[6] Noh JY Seong H Yoon JG Song JY Cheong HJ Kim WJ. Social Distancing against COVID-19: Implication for the Control of Influenza. J Korean Med Sci. 2020;35(19):e182. 10.3346/jkms.2020.35.e182 32419400 PMC7234863

[7] Sullivan SG Carlson S Cheng AC Chilver MB Dwyer DE Irwin M Where has all the influenza gone? The impact of COVID-19 on the circulation of influenza and other respiratory viruses, Australia, March to September 2020. Euro Surveill. 2020;25(47):2001847. 10.2807/1560-7917.ES.2020.25.47.2001847 33243355 PMC7693168

[8] Olsen SJ Azziz-Baumgartner E Budd AP Brammer L Sullivan S Pineda RF Decreased Influenza Activity During the COVID-19 Pandemic - United States, Australia, Chile, and South Africa, 2020. MMWR Morb Mortal Wkly Rep. 2020;69(37):1305-9. 10.15585/mmwr.mm6937a6 32941415 PMC7498167

[9] Olsen SJ Chen MY Liu YL Witschi M Ardoin A Calba C Early Introduction of Severe Acute Respiratory Syndrome Coronavirus 2 into Europe. Emerg Infect Dis. 2020;26(7):1567-70. 10.3201/eid2607.200359 32197059 PMC7323534

[10] World Health Organization. Influenza update - 388 2021. Available from: https://www.who.int/influenza/surveillance_monitoring/updates/latest_update_GIP_surveillance/en/

[11] Adlhoch C Pebody R. What to expect for the influenza season 2020/21 with the ongoing COVID-19 pandemic in the World Health Organization European Region. Euro Surveill. 2020;25(42):2001816. 10.2807/1560-7917.ES.2020.25.42.2001816 33094719 PMC7651872

[12] Cassini A Colzani E Pini A Mangen MJ Plass D McDonald SA Impact of infectious diseases on population health using incidence-based disability-adjusted life years (DALYs): results from the Burden of Communicable Diseases in Europe study, European Union and European Economic Area countries, 2009 to 2013. Euro Surveill. 2018;23(16):1700454. 10.2807/1560-7917.ES.2018.23.16.17-00454 29692315 PMC5915974

[13] Ziegler T Mamahit A Cox NJ. 65 years of influenza surveillance by a World Health Organization-coordinated global network. Influenza Other Respir Viruses. 2018;12(5):558-65. 10.1111/irv.12570 29727518 PMC6086847

[14] Mook P Meerhoff T Olsen SJ Snacken R Adlhoch C Pereyaslov D Alternating patterns of seasonal influenza activity in the WHO European Region following the 2009 pandemic, 2010-2018. Influenza Other Respir Viruses. 2020;14(2):150-61. 10.1111/irv.12703 31944604 PMC7040975

[15] Staub K Jüni P Urner M Matthes KL Leuch C Gemperle G Public Health Interventions, Epidemic Growth, and Regional Variation of the 1918 Influenza Pandemic Outbreak in a Swiss Canton and Its Greater Regions. Ann Intern Med. 2021;M20-6231. 33556268 10.7326/M20-6231 PMC7901603

[16] Petersen E Koopmans M Go U Hamer DH Petrosillo N Castelli F Comparing SARS-CoV-2 with SARS-CoV and influenza pandemics. Lancet Infect Dis. 2020;20(9):e238-44. 10.1016/S1473-3099(20)30484-9 32628905 PMC7333991

[17] European Centre for Disease Prevention and Control (ECDC) and World Health Organization (WHO) Regional Office for Europe. Operational considerations for influenza surveillance in the WHO European Region during COVID-19: interim guidance 2020. Stockholm and Copenhagen: ECDC and WHO/Europe; 2020. [Accessed 11 Feb 2021]. Available from: https://www.ecdc.europa.eu/en/publications-data/operational-considerations-influenza-surveillance-european-region-during-covid-19 and https://www.euro.who.int/en/health-topics/communicable-diseases/influenza/publications/2020/operational-considerations-for-influenza-surveillance-in-the-who-european-region-during-covid-19-interim-guidance-2020.

